# Response Surface Optimization of an Extraction Method for the Simultaneous Detection of Sulfamethoxazole and 17β-Estradiol in Soil

**DOI:** 10.3390/molecules25061415

**Published:** 2020-03-20

**Authors:** Rui Song, Qincheng Chen, Lili Yan, Pinhua Rao, Peng Sun, Lumei Wang, Guoqing Shen

**Affiliations:** 1 School of Agriculture and Biology, Shanghai Jiao Tong University, Shanghai 200240, China; SR-winter@sjtu.edu.cn (R.S.); chenqincheng@sjtu.edu.cn (Q.C.); xiaoyaopeng@sjtu.edu.cn (P.S.); wanglumei@sjtu.edu.cn (L.W.); 2 School of Chemistry and Chemical Engineering, Shanghai University of Engineering Science, Shanghai 201620, China; lily.502@163.com (L.Y.); raopinhua@hotmail.com (P.R.)

**Keywords:** antibiotics, hormone, ultrasound-assisted extraction, HPLC-UV, response surface methodology

## Abstract

Antibiotics and hormones widely exist in fertilizers and manures, which are excessively used in agriculture and animal husbandry. Considering their potential harm to the environment and human health, the detection of antibiotics and hormones has become a necessity. However, current methods find it difficult to simultaneously extract and detect antibiotics and hormones in soil and to maintain a high level of accuracy and a low cost. In this study, a straightforward, convenient, and simultaneous extraction and detection method of a representative antibiotic (sulfamethoxazole, SMZ) and hormone (17β-Estradiol, E2) in soil has been established. Ultrasound-assisted extraction (UAE) was used in the pretreatment process and high-performance liquid chromatography with the ultraviolet detector (HPLC-UV) method was then chosen in the detection process. By means of single factors and response surface experiments, optimal extraction conditions were a 41-mL buffer solution (pH 4.27) mixed with 1 g of soil sample, an ultrasonication time of 36 min, an ultrasonication temperature of 25 °C, and two extraction cycles. The detection limits of 0.3–10 μg/kg and quantification limits of 1–30 μg/kg have been obtained. Finally, the optimized simultaneous extraction and detection method was validated by three different real soil samples with recoveries ranging from 79.49% to 86.47%.

## 1. Introduction

Hormones and antibiotics are widely utilized as part of the rapid development of agriculture and animal husbandry. The incomplete adsorption of the two substances in the living body makes them excreted in the form of derived products or original structures [1,2,3,4,5,6]. These excrements can bring severe pollution in soil when used as manures, which will further have an effect on human health [7,8,9]. Sulfamethoxazole (SMZ) and 17β-Estradiol (E2) were two representative antibiotics and hormones. SMZ is a kind of sulfonamide that can be absorbed and utilized by humans and animals, applying to certain treatment of diseases with a high usage amount [10]. 17β-estradiol (E2) is a natural hormone that is linked to fish feminization near treatment plant outfalls [11]. As one of the estrogens with excellent activity, E2 can be metabolized to estrone and estriol. In a soil environment, E2 can organically combine with soil particles, leading to sequential accumulation in soil [12,13,14]. In fact, as co-pollutants with steroid hormones in animal waste, SMZ and E2 generally simultaneously exist in the soil near the farms [15]. Thus, the simultaneous detection of these two substances matters greatly [16]. However, previous work concentrated little on simultaneous pretreatment and detection methods of SMZ and E2 in soil because of difficult extraction and poor detection specificity [17,18,19,20,21].

Sample pretreatment, including ultrasound-assisted extraction (UAE) [16,22], supercritical fluid extraction (SFE) [23], liquid–liquid extraction (LLE) [23], pressurized liquid extraction (PLE) [24], and solid phase extraction (SPE) [16], is one of the key factors related to the determination of SMZ and E2 in soil because of matrix interference effects [25]. However, the utilization of PLE, SFE, and LLE in soil detection was still restricted by high cost and consumption. In addition, SPE is usually used for concentrating samples [26,27], but in order to simplify the process, some researchers explored other pretreatment methods without SPE [28,29]. UAE is considered as a general pretreatment method in soil with high experimental convenience, low cost, and less reagent consumption, which has good potential in sample pretreatment.

In practical applications, several factors, including solution pH, volume in pretreatment, and the detection method, have a crossed influence on the recovery results together, so simultaneous optimization of them is significant [30]. Single-factor experiments are usually used in conventional multivariable optimization to detect independent variables without presenting interactions among all determinants [31]. Response surface methodology (RSM) is a data analysis method to investigate and optimize the factors at the same time, which can design the most reasonable experimental scheme according to the actual demand [32]. Excellent predictability, simple operation, and high efficiency make RSM widely applied in biotechnology and food processing industries [33,34,35].

As the concentration of antibiotics and hormones is quite low in soil, high precision and specificity and a low detection limit are essential for detection methods [36]. Many studies have employed detection methods using high performance liquid chromatography-mass spectrometry (HPLC-MS) [37] or liquid chromatography-mass spectrometry (LC-MS) [16] for some common antibiotics and hormones including SMZ and E2. However, the high cost and complex operation cannot be ignored [19]. In order to simplify the detection process and reduce the cost [38], HPLC equipped with an ultraviolet detector was chosen for simultaneous SMZ and E2 detection in this work.

Herein, we innovatively established a simple, efficient, and simultaneous pretreatment method for SMZ and E2 with HPLC-UV detection. The conditions, including ultrasonication time, solution pH, and volume in UAE, were investigated using single-factor and RSM optimization. The optimal method for the two representative antibiotic and hormone was then validated in real soil sample, which can be extended to simultaneous extraction and detection of similar substances.

## 2. Results and Discussion

### 2.1. Determination of the Conditions for the HPLC Detection Method

In this study, 274 [36] and 280 nm [39], with the strongest UV absorption peaks of SMZ and E2 correspondingly, were selected as the possible detection wavelengths. Comparing the chromatograms of same standard solution in two wavelengths, hence 280 nm was chosen for further detection due to a better response of E2 than that of 274 nm.

**Figure 1:**
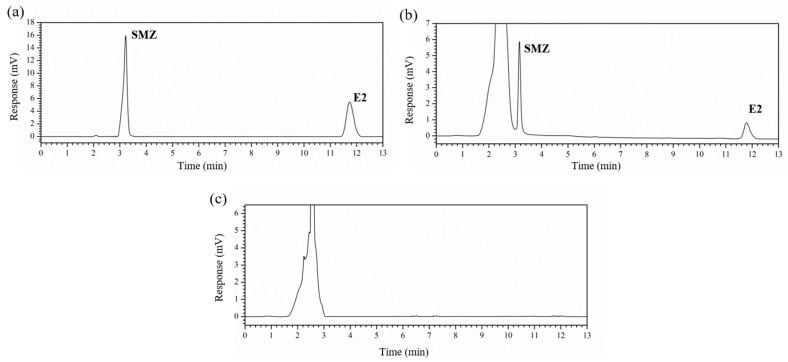
Chromatograms obtained by optimized detection conditions of (**a**) standard solutions of 500 μg/kg sulfamethoxazole (SMZ) and 17β-Estradiol (E2) in methanol, (**b**) the extraction solution of the blank soil sample with 20 mg/kg SMZ and E2, and (**c**) the blank soil sample extract.

The mobile phase, flow rate, column temperature, and other factors were tested in the following experiments as well. Methanol/water (70:30, *v*/*v*) was chosen as the mobile phase [40]. The flow rate of 1.0 mL/min can not only ensure the detection efficiency, but also avoid the peak accumulation, which was also selected. The column pressure can be easily controlled and maintained at a stable level at the column temperature of 30 °C. Additional detection conditions have been conducted by a C18 column and binary high-pressure elution with 10 μL per injection. As shown in Figure 1, legible and interference-free peaks of SMZ and E2 exhibited detectability, with invariable retention times of 3.2 and 11.8 min, respectively.

To explore the practicability of the detection method, a series of gradient concentration standard solutions have been prepared and detected. Standard liner fitting curves of SMZ and E2 were then established (Table 1). In the concentration range of 30–10,000 μg/kg, the peak response showed linear fitting via a linearity index (R^2^) of 0.99998 and 0.99991 for SMZ and E2, which indicated a feasible simultaneous detection method for the two substances. Generally speaking, the value of LOD (Limit of Detection) and LOQ (Limit of Quantitation), determining to be the lowest accomplishable concentrations with signal-to-noise ratios of 3 and 10 [41], can estimate the accuracy and precision of the detection method. In our detection system, the values of LOD were 0.3 μg/kg and 10 μg/kg, with the LOQ of 1 μg/kg and 30 μg/kg as well for SMZ and E2, respectively. This that a meant comparable detection limit was obtained that can be used in trace detection.

### 2.2. Single Factor Tests of Ultrasonic Extraction Pretreatment

In order to better study the crossed influence of multiple factors by RSM method, we firstly carried out single-factor experiments to select the conditions with obvious influence on the recoveries.

#### 2.2.1. Volumes of Extraction Solution

**Figure 2:**
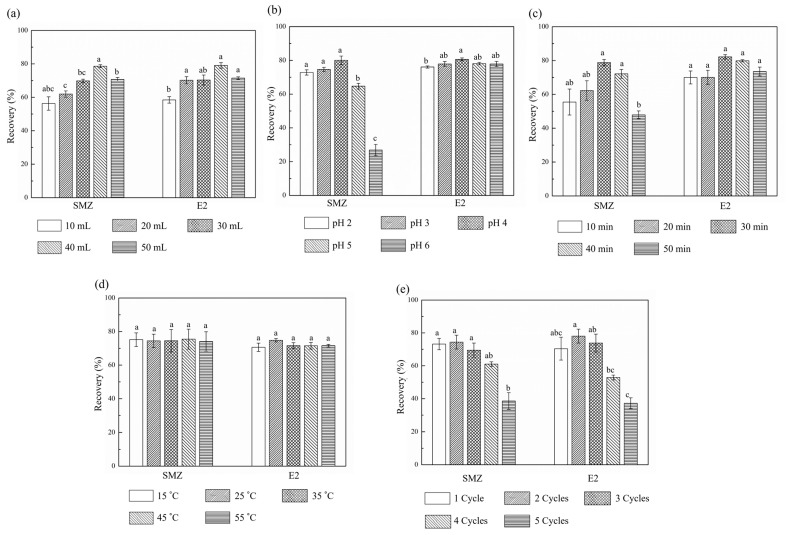
Influence of solution volume (**a**), pH (**b**), ultrasonication time (**c**), temperature (**d**), and extraction cycles (**e**) on SMZ and E2 recovery rates. Different letters (a, b, c) show statistically significant different means (*P* < 0.05).

As can be seen from Figure 2a, the extraction recovery rates of SMZ and E2 increased first and then decreased with the highest recovery values (78.15% and 80.92%) appearing in a total volume of 40 mL. The appropriately increased volume can distinctly lead to additional solution with a higher extraction capacity. However, the raised dispersion degree of contaminants with a decreased concentration could be caused by the excessively high volume, resulting in deterioration in detection. Since the volume variation had significant influence on the average extraction recovery rates, the range of 30–50 mL near the highest value was selected for further RSM optimization.

#### 2.2.2. pH Values of Extraction Solution

Figure 2b shows the trends of recovery rates accompanied with the variation of pH values from 2 to 6. The extraction recovery rates of E2 remained at a quite high (more than 75%) and stable level without the obvious influence of pH effect because the alcoholic hydroxyl group in E2 structure can maintain high stability under acidic condition [12]. Nevertheless, that was not the case for SMZ due to an amphoteric structure [42], which reached distinct variation surpass 50% when the pH values changed. Within the scope of 3 to 5, most of the SMZ molecules formed a neutral steady state. So, at this point, SMZ can be extracted more efficiently and stably. The highest recovery rate (82.31%) of SMZ was obtained at the pH value of 4. Therefore, a range of pH values from 3 to 5 was selected for RSM optimization.

#### 2.2.3. Ultrasonication Time

Similar to the extraction solution volume, the recovery rates of SMZ and E2 varied significantly with an increasing ultrasonication time from 10 to 50 min (Figure 2c). As the total time increased, the structures of the soil samples broke easily, which led to raised recoveries. However, when the ultrasonic time was long, the obtained crude extract could be adsorbed back to the soil with the influence of soil–water partition coefficients, resulting in decreased recoveries [43,44]. This declining trend may also be because the prolonged ultrasonic time can degrade the extraction targets or accompany the dissolution of other impurities [45]. The highest recoveries (76.50% and 83.68%) appeared at 30 min. Therefore, an ultrasonication time of 20, 30, and 40 min was chosen for subsequent experiment.

#### 2.2.4. Ultrasonication Temperature

It can be seen from Figure 2d that when the ultrasonication temperature increased from 15 to 55 °C, the fluctuation extent of recoveries was less than 5%, indicating an inconspicuous influence of ultrasonication temperature on the recovery rates of SMZ and E2. Therefore, 25 °C, with the highest recovery rates of 73.70% and 76.51%, was selected as the ultrasonication temperature in the following experiments.

#### 2.2.5. Total Cycles of Extraction

As demonstrated in Figure 2e, the highest recoveries of 74.39% and 78.06% appeared with two cycles of extraction. More substances can be extracted with better dispersion when the extraction cycles increased. Nevertheless, further extraction cycles can lead to a greater loss of extraction and lower recoveries at the same time. Since the number of extraction cycles must be an integer, this factor need not be optimized for RSM, and extraction cycles were set as two.

### 2.3. RSM Optimization

Based on forgoing the results of single-factor experiments, the ultrasonication time (X_1_) and the pH values (X_2_) and volume (X_3_) of the extraction solution were selected for RSM optimization. The optimized conditions obtained from the software, Design-expert 8.0, and the corresponding experimental results were shown in Table 2. The average recoveries of SMZ and E2 ranged between 62.71% and 81.30%.

The average recovery was taken as the dependent variable Y with ultrasonication time (X_1_), and the pH value (X_2_) and volume (X_3_) of the extraction solution were chosen as the independent variables. The fitted quadratic regression model obtained was as follows:

Y = 81.20 + 1.25 X_1_ + 2.60 X_2_ − 0.65 X_3_ + 2.24 X_1_X_2_ + 1.47 X_1_X_3_ + 1.69 X_2_X_3_ − 1.64 X_1_^2^ − 7.49 X_2_^2^ − 5.85 X_3_^2^. (1)

Variance analysis of the fitted model was shown in Table 3, which was acquired from Design-expert 8.0 automatically. For the significance analysis, the *P* value of the quadratic model was 0.0029, less than 0.01, suggesting that it was an extremely significant model with better fitting of the experimental data. The lack of fit *P* value was 0.0691, so the miscalculation was not significant and the model exhibited great applicability. The determination coefficient (*R*^2^) and adjusted determination coefficient (Adj *R*^2^) were 0.9689 and 0.9128, correspondingly, which showed little difference between actual and fitted data, also identifying the accurate prediction of the model. To summarize, among the three independent variables, the pH value of the extraction solution (X_2_) had significant effect on recoveries. The interactive influence of ultrasonication time and pH (X_1_ and X_2_) was significant, as well.

**Figure 3:**
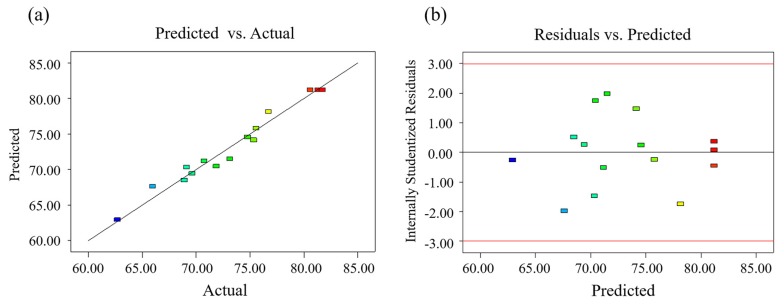
Obtained response of predicted and actual values (**a**), and the relationship between internally studentized residuals and predicted recoveries (**b**).

The relationship between predicted and actual recoveries was shown in Figure 3a. All actual recovery points were near the predicted fitting line, which suggested excellent correlation between actual and predicted response. Besides, corresponding to each predicted recovery shown in Figure 3b, all residual points stayed stable within a range of [−2.00, +2.00] randomly. Hence, it was stable for the variance obtained from RSM experiments to every Y value.

**Figure 4:**
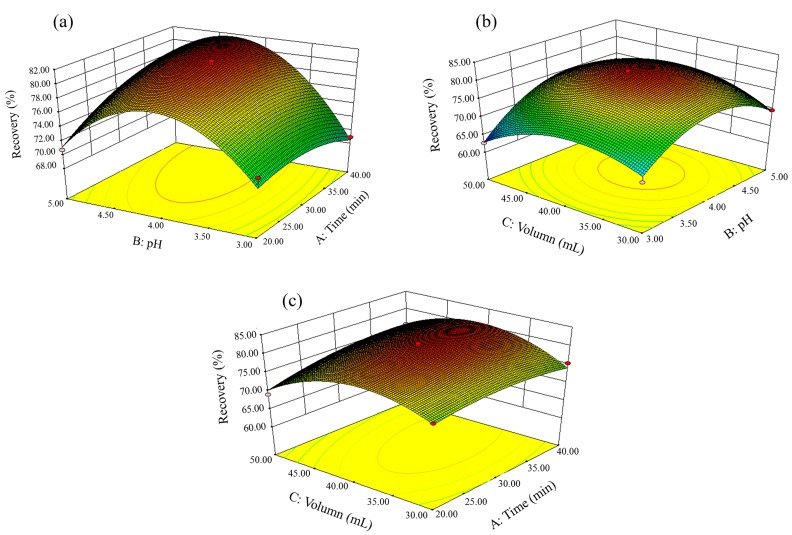
Response surfaces for interactive effects of (**a**) pH value of solution versus ultrasonication time (solution volume: 30 mL), (**b**) pH value versus volume of solution (ultrasonication time: 30 min), and (**c**) volume of solution versus ultrasonication time (pH value of solution: 4).

In order to investigate the interactive effects of every two factors more intuitively, 3D response surface graphs were explored in Figure 4. It showed that the effects of the remaining variables to the Y value when one of the variables was maintained at a 0-coding level (the middle of the variation range). Generally, small or no interaction between two factors indicates a flat response surface. On the contrary, obvious interaction will lead to a curved response surface. As can be seen from Figure 4a, with a fixed solution volume of 30 mL, recoveries (Y) increased as the pH rose value from 3.0 to 4.1 and then decreased in a much higher pH value. The ultrasonication time also had positive effect on recoveries with improved response. The obvious curved response surface in Figure 4a indicated that it is significant for the interaction between pH value and ultrasonication time. Figure 4b,c reflected the response surfaces of solution volume versus pH and ultrasonication time, respectively. However, corresponding to Table 3, the flat surfaces illustrated insignificant (*P* > 0.05) interaction between two factors.

In this part, with the help of RSM, extraction conditions using ridge maximum analysis [44] were optimized, which can generate an increased radius from the center of the original design for maximum response estimation. The RSM model predicted that the maximum average recoveries of SMZ and E2, which can reach 81.90%, would appear at the conditions of 36 min for ultrasonication time, 4.27 for pH value, and 41 mL for extraction solution volume.

### 2.4. Method Validation for Real Samples

The optimal extraction conditions for maximum recoveries of SMZ and E2 have been obtained by RSM experiment. In order to verify the conditions, three actual soil samples were collected from Songjiang, Minhang, and Qingpu district, respectively, in Shanghai. The contents of SMZ and E2 in these soil samples were below the LOD of our HPLC detection method. Thus, SMZ and E2 standards of 20, 40, and 60 mg/kg were added into blank soil samples for validation. All polluted soil samples were then extracted in optimal conditions. The individual and average recoveries of SMZ and E2 were shown in Table 4.

It can be seen from Table 4 that the average recoveries were 81.78%, 82.02%, and 85.05% for the polluted soil samples of 20, 40, and 60 mg/kg, respectively, which were consistent with the predicted average recovery in RSM (81.90%) with a tiny deviation of less than 6%. Moreover, all recoveries of SMZ and E2 ranged from 74.67% to 89.32%. RSD within 3.55% demonstrated strong practical applicability of the optimal extraction conditions obtained from RSM. Therefore, the proposed method can be used for the simultaneous extraction of SMZ and E2 in actual soil samples.

### 2.5. Comparison with Other Existing Methods

In current studies, LC-MS and HPLC-MS are generally used in the detection of antibiotics and hormones in soil, but the complicated operation or longer detection time (25–53 min) of these methods cannot be ignored [16,22,37,46]. In this study, HPLC-UV was chosen as the detection technique and can significantly shorten the detection time to 15 min. Furthermore, comparable LODs for SMZ [16,37] and E2 [37,47] have been obtained. Moreover, in contrast to other extraction methods, less time by 10–60 min was taken in our proposed method [16,22,37,46,47], which is mainly due to the optimization of extraction conditions. The clean-up and enrichment steps including SPE and MIP were also left out, which can further shorten the extraction time. In particular, after simultaneous extraction and detection, recoveries of 74.67%–89.32% were achieved in our work, which well met the extraction recovery range of 70% to 120% recommended by the United States Environmental Protection Agency (US EPA) [48]. In short, this proposed method can be considered to be a straightforward, rapid, convenient, efficient, and low-loss way for the simultaneous extraction and detection of SMZ and E2 in soil.

## 3. Materials and Methods

### 3.1. Materials and Reagents

The sulfamethoxazole (SMZ, 99.6%) used in the experiments was purchased from Energy Chemical (Shanghai, China). The 17β-Estradiol (E2, 99.9%) was purchased from Aladdin (Shanghai, China). Methanol (HPLC grade, 99.6%) and acetonitrile (HPLC grade, 99.6%) were obtained from ANPEL Laboratory Technologies Inc. (Shanghai, China). Other analytical reagents were purchased from J&K Chemical. Technology Co., Ltd. (Shanghai, China). The soil samples (0–20 cm) were collected from the farmland near the School of Agriculture and Biology, Shanghai Jiao Tong University (Shanghai, China). The soil was composed of 57.00% clay, 24.10% silt, and 18.90% sand. The concentration of total carbon, total nitrogen, total phosphorus, and pH in the soil was 15.00g/kg, 1.10 g/kg, 0.10 g/kg, and 5.83, respectively. All the presented recoveries in the manuscript were obtained from the analysis of fortified soil samples.

### 3.2. Sample Processing

Excavated soil samples were grounded after being air-dried for two weeks. Then, a 100-mesh sieve was used to filter them for making the added pollutants more uniform in the artificial soil samples. Prior to adding SMZ and E2, the filtered soil samples were sterilized at 121 °C three times for 45 min.

Standard solutions (10 mg/L) of SMZ and E2 were prepared by dissolving them into methanol. The sterilized soil samples 100 g were spiked with 200 mL of standard solutions that contained SMZ and E2, and an initial concentration of 20 mg/kg for the processed soil samples was obtained. Then, the soil samples were dried at room temperature for at least 24 h for further experiments.

### 3.3. Detection Conditions with HPLC

SMZ and E2 were determined by HPLC with an ultraviolet detection system (Shimadzu SPD-20A, Tokyo, Japan). A Syncronis C18 250 × 4.6 × 5 μm column (Thermo Fisher Scientific, Shanghai, China) was selected as the separation column. The column temperature was set at 30 °C. The binary high-pressure elution mode was chosen with a flow rate of 1.0 mL/min and an injection volume of 10 μL. The mobile phase was methanol/water (70:30, *v*/*v*) and the UV detection wavelength was 280 nm. All samples needed to be filtered by a 0.22-μm filter (hydrophilic PTFE syringe filter, Anpel, Shanghai, China) before HPLC testing.

### 3.4. Extraction Method

The basic method for extracting SMZ and E2 from soil was as follows: 1 g of processed soil was placed in a 50-mL centrifuge tube with the extraction buffer. The extraction buffer was prepared by mixing methanol, citrate buffer solution (the ratio of citric acid and sodium citrate solution determined the pH of the extraction solutions) and 0.1 mol/L Na_2_EDTA aqueous solutions at a ratio of 3:2:1 (*v*/*v*/*v*), which was frequently reported as an extraction solution for extracting antibiotics in soil [19,49]. After homogenizing for 1 min, the tube was ultrasonicated in an ultrasonic bath (SB-5200-DTD, Ningbo, China) for a period of time under certain temperature conditions. The type, dosage, and pH of the extraction buffer, the ultrasonic temperature, time, and the number of sonication cycles were all determined by single-factor experiments. After the ultrasound, extraction solution was centrifuged (Thermo Scientific SL-16 Centrifuge, Shanghai, China) at 4000 r/min for 5 min. The supernatant was taken and filtered by a 0.22-μm filter, the final extracting solution was placed in a 1.5-mL brown sample for HPLC testing.

### 3.5. Single-Factor Experiments

The volume of the extraction buffer (10, 20, 30, 40, and 50 mL), the pH of the extraction solution (3, 4, 5, 6, and 7), the ultrasonic temperature (15, 25, 35, 45, and 55 °C), ultrasonic time (10, 20, 30, 40, 50 min), and the frequency of extraction (1, 2, 3, 4 and 5) were explored in this part. The effect of the single factor was tested with all other factors kept constant, and the experimental results were repeated three times. SPSS Statistics 17.0 was chosen to analyze the recovery rates in different extraction conditions. The significant influence of the single factor can be indicated when the *P* value was below 0.05. Three factors with the most obvious influence were selected for subsequent response surface optimization.

### 3.6. Response Surface Optimization (RSM)

According to the single-factor experimental results of the extraction method, the ultrasonic time (X_1_), the pH of the extraction solution (X_2_), and the total volume of the extraction buffer (X_3_) were selected as the optimized factors in the RSM experiments. The dependent variables (Y) here were the average recovery rates of SMZ and E2. Design-Expert.V.8.0.6 was used to design a 15-run Box-Behnken (BBD) experiment. Here, a wildly used quadratic regression model was selected [50], and the corresponding formula is as follows:

Y = A_0_ + ∑ A_i_X_i_ + ∑ A_ii_X_i_^2^ + ∑ A_ij_X_i_X_j_. (2)

In the formula, Y is the predicted value obtained from the RSM method. A_i_, A_ii_, and A_ij_ refer to the linear coefficient, the quadratic term coefficient, and the coefficient of interactive terms, respectively. X_i_ and X_j_ represent the independent variables in the model. The obtained results from the RSM model were analyzed by Design-Expert V.8.0.6. (StatEase, Minneapolis, MN, USA) When the *P* value of the model was less than 0.05, it can be indicated that the obtained model is significant [51].

### 3.7. Method Validation

The optimal extraction conditions that maximize the average recovery rate of SMZ and E2 were obtained from the RSM optimization. In order to verify whether the obtained model has practical application, verification experiments were conducted in this part.

Three soil samples were collected from different place for extraction and HPLC detection. However, the amounts of SMZ and E2 in the samples did not reach the detection limit of the optimized method, so 20, 40, and 60 mg/kg pollutant standards were added into the blank samples for verification experiments. These soil samples were pretreated using the optimized extraction method from the RSM experiment, and three sets of parallel experiments were conducted, respectively. The slight difference between the actual recovery rates and the predicted maximum recovery rate indicates that the model obtained from the RSM experiment has practical application, and the optimized extraction recovery has great repeatability and reference.

## 4. Conclusions

In this paper, a representative antibiotic (SMZ) and hormone (E2) have been selected to establish a simultaneous pretreatment and detection method in soil. UAE was used in the pretreatment process and HPLC-UV was chosen in detection. Single-factor experiments and RSM were then used to optimize the extraction methods. According to the single-factor experiments, ultrasonication time and the pH value and volume of the extraction solution had a significant influence on average recoveries and were further optimized for RSM. After RSM optimization, optimal extraction conditions, including an ultrasonication time of 36 min, a solution pH value of 4.27, and a volume of 41 mL, have been acquired with a predicted average recovery of 81.90%. This optimized method was then applied in three types of actual soil samples, and the results showed recoveries ranging from 79.49% to 86.47% with low RSD (<3.55%). In addition, low LODs (0.3 and 10 μg/kg) and LOQs (1 and 30 μg/kg) of the detection method have also been obtained. In all, we have established a more straightforward, convenient, and efficient method for the simultaneous detection of SMZ and E2 in soil, which can be extended to a wide range of applications in contamination detection.

## Figures and Tables

**Table 1:** Regression equations and detection limits.

Compound	Linear Regression Equation	R^2^	LOD (μg/kg)	LOQ (μg/kg)
SMZ	Y = 33.696X + 2115.651	0.99998	0.3	1
E2	Y = 4.367X + 60.425	0.99991	10	30

**Table 2:** Response surface methodology (RSM) optimization design and results of the average recoveries of SMZ and E2.

	X_1_	X_2_	X_3_	Y
	Ultrasonication Time (min)	pH Values	Volume (mL)	Recovery (%)
1	20.00 (−1)	4.00 (0)	50.00 (+1)	69.12
2	30.00 (0)	4.00 (0)	40.00 (0)	81.70
3	30.00 (0)	3.00 (−1)	30.00 (−1)	65.97
4	30.00 (0)	5.00 (+1)	30.00 (−1)	69.64
5	30.00 (0)	5.00 (+1)	50.00 (+1)	73.13
6	40.00 (+1)	3.00 (−1)	40.00 (0)	68.91
7	20.00 (−1)	4.00 (0)	30.00 (−1)	74.78
8	20.00 (−1)	5.00 (+1)	40.00 (0)	70.75
9	40.00 (+1)	5.00 (+1)	40.00 (0)	76.72
10	40.00 (+1)	4.00 (0)	50.00 (+1)	75.57
11	30.00 (0)	4.00 (0)	40.00 (0)	80.59
12	40.00 (+1)	4.00 (0)	30.00 (−1)	75.36
13	30.00 (0)	3.00 (−1)	50.00 (+1)	62.71
14	20.00 (−1)	3.00 (−1)	40.00 (0)	71.88
15	30.00 (0)	4.00 (0)	40.00 (0)	81.30

**Table 3:** Variance analysis of the response surface fitted model.

Source	Sum of Squares	Df ^a^	Mean Square	*F*-value ^b^	*P* value ^c^
Model	421.87	9	46.87	17.29	0.0029
X_1_, time (min)	12.58	1	12.58	4.64	0.0839
X_2_, pH	53.92	1	53.92	19.89	0.0066
X_3_, volume (mL)	3.41	1	3.41	1.26	0.3133
X_1_X_2_	19.98	1	19.98	7.37	0.0421
X_1_X_3_	8.61	1	8.61	3.18	0.1348
X_2_X_3_	11.39	1	11.39	4.20	0.0957
X_1_^2^	9.97	1	9.97	3.68	0.1133
X_2_^2^	207.05	1	207.05	76.35	0.0003
X_3_^2^	126.18	1	126.18	46.53	0.0010
Residual	13.56	5	2.71		
Lack of fit	12.93	3	4.31	13.63	0.0691
Pure error	0.63	2	0.32		
*R* ^2^	0.9689				
Adj *R*^2^	0.9128				

^a^ Degree of freedom; ^b^ Test for comparing model variance with residual variance; ^c^ The probability of seeing the observed *F*-value if the hypothesis is true.

**Table 4:** Validation results of optimal extraction conditions.

Added (mg/kg)	Soil Samples ^a^	SMZ Recovery (%)	E2 Recovery (%)	Average Recovery (%)	Integral Average Recovery (%)	RSD ^b^ (%)
20	S1	84.85 ± 0.81	81.26 ± 0.83	83.06 ± 0.68	81.78 ± 1.62	1.62
	S2	82.32 ± 2.63	76.66 ± 0.97	79.49 ± 0.85		
	S3	87.51 ± 1.66	78.07 ± 2.90	82.79 ± 1.81		
40	S1	78.79 ± 2.96	86.06 ± 2.01	82.42 ± 4.81	82.02 ± 0.25	0.24
	S2	74.67 ± 1.29	89.32 ± 1.81	81.99 ± 1.51		
	S3	76.54 ± 1.90	87.14 ± 0.46	81.84 ± 0.74		
60	S1	80.66 ± 2.35	88.27 ± 1.53	86.47 ± 0.57	85.05 ± 1.00	3.55
	S2	81.72 ± 1.74	86.86 ± 0.92	84.29 ± 0.86		
	S3	82.44 ± 1.31	86.35 ± 1.55	84.40 ± 0.36		

^a^ Soil samples collected from Songjiang (S1), Minhang (S2), and Qingpu (S3) District in Shanghai, P. R. China, respectively; ^b^ RSD: Relative standard deviation.
